# Pyloric adenomatous carcinoma of the gallbladder following laparoscopic cholecystectomy: A case report

**DOI:** 10.1016/j.ijscr.2021.106278

**Published:** 2021-08-05

**Authors:** Nao Kitasaki, Tomoyuki Abe, Akihiko Oshita, Keiji Hanada, Toshio Noriyuki, Masahiro Nakahara

**Affiliations:** Department of Surgery, Gastroenterology, Onomichi General Hospital, Onomichi, Hiroshima, Japan

**Keywords:** CT, computed tomography, GBC, gallbladder cancer, HGM, human gastric mucin, CEA, carcinoembryonic antigen, CA19-9, carbohydrate antigen 19-9, US, ultrasonography, MRCP, magnetic resonance cholangiopancreatography, EUS, endoscopic ultrasound, LC, laparoscopic cholecystectomy, DL-DSS, deep learning-based decision support system, AI, artificial intelligence, Incidental gallbladder cancer, Gallbladder adenoma, Laparoscopy, Surgery

## Abstract

**Introduction:**

Adenoma and intra-adenoma carcinoma of the gallbladder are relatively rare diseases, and the World Health Organization classification reports a frequency of 0.3% for gallbladder adenomas. Precise preoperative diagnosis of gallbladder cancer, especially in the early stages, is challenging. Herein, we report a case of pyloric adenomatous carcinoma of the gallbladder, diagnosed by laparoscopic cholecystectomy and pathology, along with a literature review. This case was reported in accordance with the SCARE 2020 Guideline (Ref).

**Presentation of case:**

A 62-year-old woman was diagnosed with a 4-mm polypoid lesion in the gallbladder during a medical examination. The patient was followed-up by ultrasonography (US) once a year and was referred to our department because of an increase in size. Carcinoembryonic antigen and carbohydrate antigen 19-9 levels were within normal limits. Abdominal ultrasonography revealed a pedunculated polypoid lesion in the body of the gallbladder measuring 8 mm. Computed tomography demonstrated that the whole tumor was enhanced in the early phase without significant lymph node enlargement. Magnetic resonance cholangiopancreatography demonstrated a type Ip polypoid lesion located in the body of the gallbladder without pancreaticobiliary junctional abnormalities. Endoscopic ultrasound detected a superficial nodular-type Ip polypoid lesion in the gallbladder body with a parenchyma-like internal echogenic pattern.

**Discussion:**

Based on these findings, the patient was diagnosed with gallbladder adenoma, and laparoscopic cholecystectomy was performed. Histopathological examination revealed the tumor was a papillary growth of atypical high columnar epithelial cells. The final diagnosis was pyloric adenoma with high-grade dysplasia and intra-adenoma carcinoma. The patient is currently undergoing outpatient follow-up without recurrence for 1 year.

**Conclusion:**

Early gallbladder carcinoma with adenoma should be considered in patients with small gallbladder polypoid lesions. Considering the surgical stress of cholecystectomy and the malignant potential of gallbladder cancer, preceding surgery would be acceptable.

## Highlights

• Incidental gallbladder cancer is rare and preoperative assessment of GBPL is important.
• Considering the malignant potential of GBC, preceding surgery would be acceptable.

## 1 Introduction

Gallbladder cancer (GBC), one of the top six gastrointestinal tract neoplasms worldwide, is the most common malignancy of the biliary tract [1], [2]. Precise preoperative diagnosis of early stage GBC is challenging. Incidental GBC is found preoperatively following cholecystectomy in approximately 1% of patients with benign gallbladder disease [3]. The prognosis of GBC even after curative surgery is poor, with a 5-year overall survival rate of approximately 20% [4], [5]. To date, perioperative systemic chemotherapy has not been established.

Adenomas and small GBCs with intracholecystic papillary-tubular neoplasm, gastric pyloric, and simple mucinous types are rare. The World Health Organization (WHO) classification states that the frequency of gallbladder adenomas is approximately 0.3% [6]. Various factors such as tumor size, lymph node metastasis, perineural invasion, and TNM stage have been reported as significant prognostic factors after curative resection of GBC [7], [8]. The adenoma-carcinoma sequence is a well-known pathway of carcinogenesis, and optimal surgical timing is essential for a good prognosis. In general, a polyp diameter of 10 mm is the cut-off for determining the treatment strategy, and a larger diameter is strongly related to a higher incidence of adenocarcinoma [5], [9].

Herein, we report the case of a patient with a small GBC with intracholecystic papillary-tubular neoplasm, gastric pyloric and simple mucinous type, after laparoscopic surgery. A literature review is also presented.

## 2 Case presentation

**Fig. 1:**
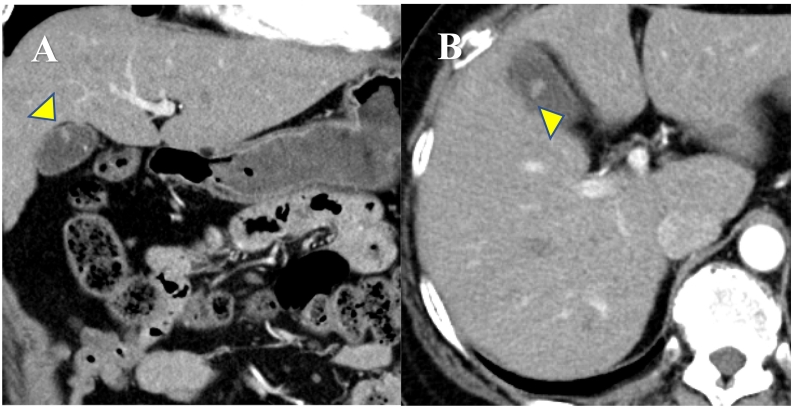
Abdominal computed tomography findings. A B: The arterial phase showing a slightly enhanced low-density tumor, 8 mm in size, located at the bottom of the gallbladder.

**Fig. 2:**
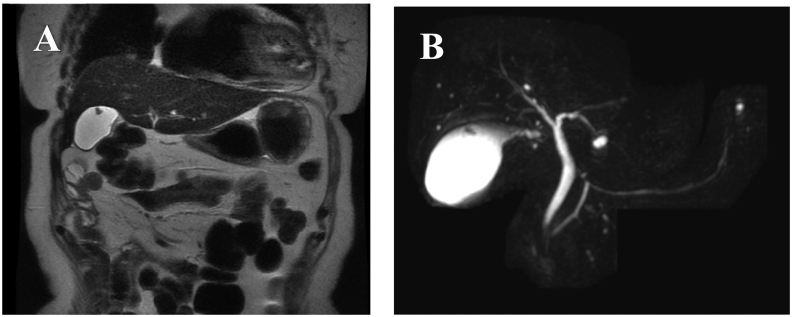
Findings of magnetic resonance imaging (MRI): A: The tumor shows a low signal intensity on a T2-weighted image. B: T2-weighted MRCP image showing a pedunculated tumor located at the bottom of the gallbladder.

**Fig. 3:**
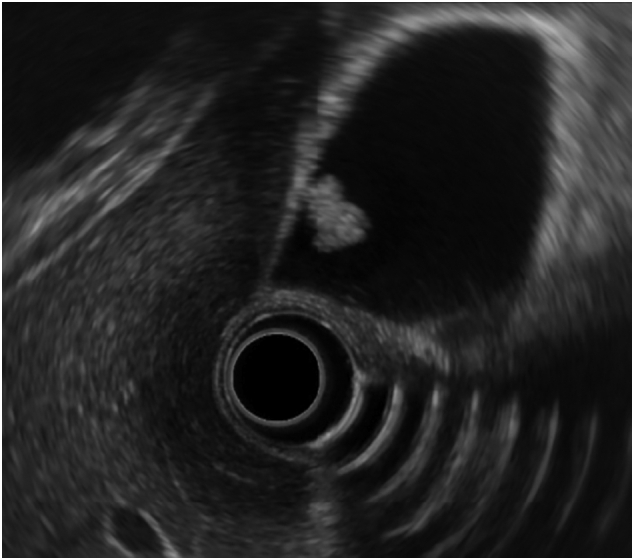
Endoscopic ultrasound (EUS) showing a hyper echoic pedunculated shaped tumor 8 mm in size located in the bottom of the gallbladder with a parenchymal-like internal echogenic pattern.

**Fig. 4:**
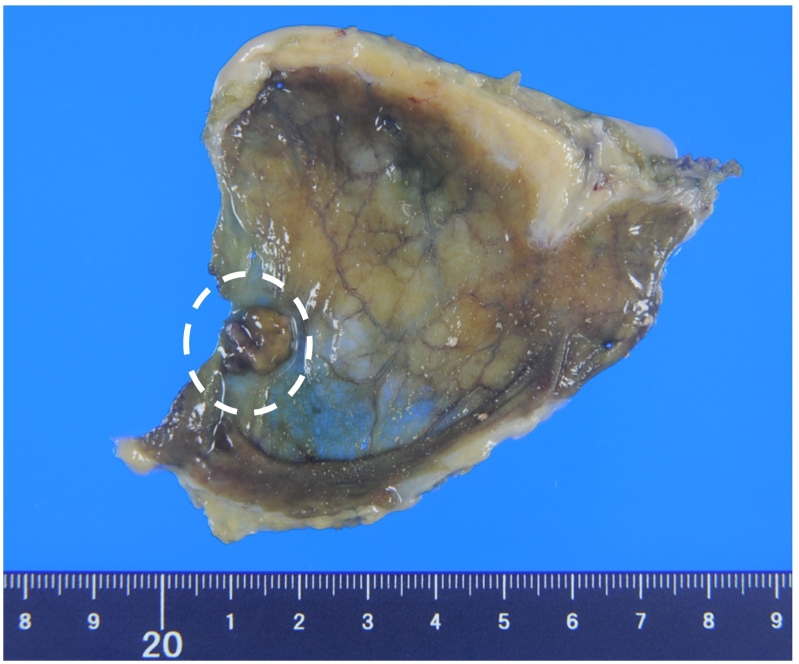
Imaging: Resected specimen showing yellow pedunculated (type 1p) tumor 10× 10 mm in size located in the bottom of the gallbladder. (For interpretation of the references to color in this figure legend, the reader is referred to the web version of this article.)

**Fig. 5:**
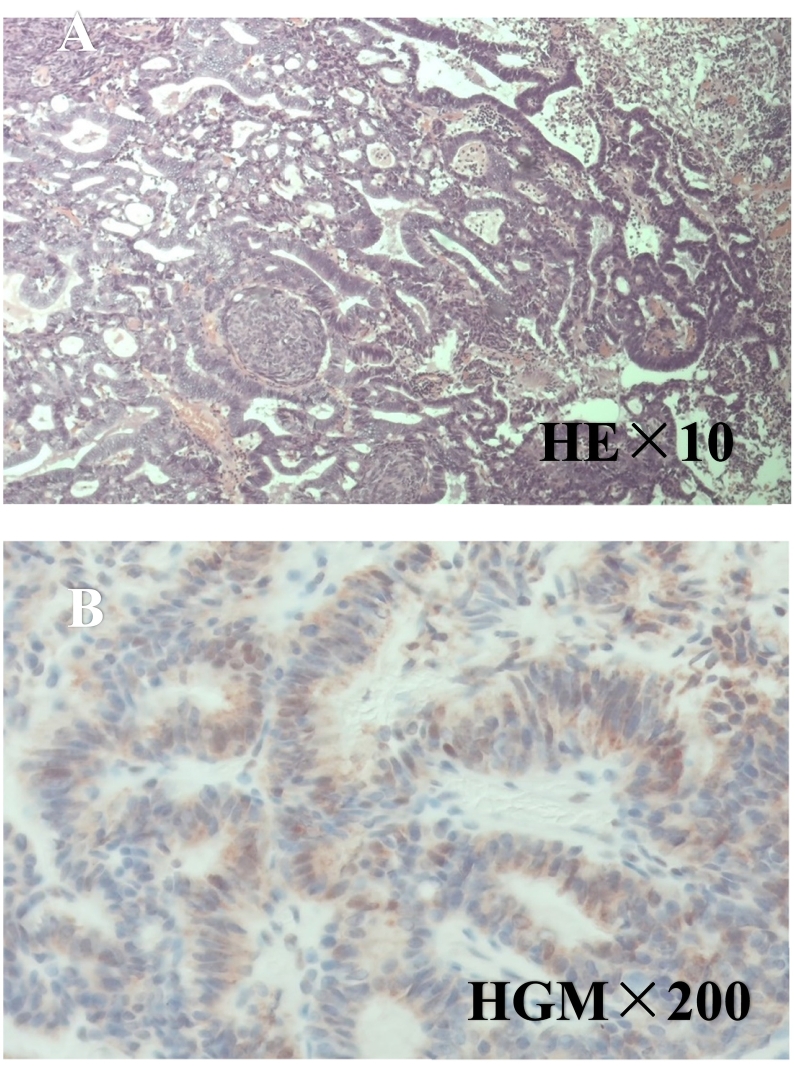
A: The tumor consists of papillary growth of atypical high-columnar epithelial cells. The tumor contains a microcarcinoma in the adenoma. B: The tumor is positive for human gastric mucin (HGM).

A 62-year-old woman was diagnosed with a 4-mm polypoid lesion in the gallbladder during a medical examination in 2014. The patient was followed-up by ultrasonography (US) once a year and was referred to our department because of an increase in size of 4 mm in 6 years. Her medical history included a duodenal ulcer and primary biliary cirrhosis. Blood examinations showed that carcinoembryonic antigen (CEA) and carbohydrate antigen 19-9 (CA 19-9) levels were within normal limits. Abdominal ultrasonography showed a polyp measuring 8 mm with a stalk, in the body of the gallbladder, and computed tomography revealed that there was no significant lymph node enlargement around the hepatoduodenal ligament (Fig. 1A, B). Magnetic resonance cholangiopancreatography (MRCP) revealed a type Ip polypoid lesion in the gallbladder body (Fig. 2A, B). Endoscopic ultrasound (EUS) demonstrated that this lesion represented a parenchyma-like internal echogenic pattern (Fig. 3). Based on these findings, the patient was diagnosed with gallbladder adenoma, and laparoscopic cholecystectomy (LC) was performed. The intraoperative time was 101 min, and blood loss was 5 mL. Intraperitoneal bile leakage was not observed. The excised tissue was a yellowish, pedunculated (type Ip) tumor (Fig. 4) measuring 10 mm. On histopathological examination, the tumor showed papillary growth of atypical tall columnar epithelial cells. On immunohistological analysis, the tumor was positive for human gastric mucin (HGM) and negative for Alcian blue, CD10, villin, and CDX2. The mitotic index was <1%, MIB-1 positive (3%), localized p53 positive, mucin expression MUC5AC positive, MUC1 weakly positive, and MUC2 negative. The final diagnosis was gallbladder carcinoma in situ with intracholecystic papillary-tubular neoplasm, gastric pyloric, and simple mucinous type with high-grade dysplasia (Fig. 5A, B). The patient is currently undergoing outpatient follow-up without any postoperative recurrence for one year.

## 3 Discussion

With the improvement of diagnostic interventions, such as EUS, small-sized gallbladder tumors such as gallbladder polypoid lesions (GBPLs) have been precisely detected. Malignancy is a widely known characteristic feature of small GBPLs [10]. GBPLs are classified into papillary, nodular, flat, filled, and lumpy types based on their gross morphology. The possibility of early stage cancer is high in tumors with papillary morphology, as in our case. Various studies have been conducted on the morphological characteristics of GBPLs in terms of size, number, and internal echogenicity [11], [12]. However, distinguishing GBPLs from GBC remains challenging [10], [11], [13].

Determining the optimal surgical timing for GBPLs is essential to prevent cancer development while avoiding unnecessary surgery. An accepted cut-off for surgery is a tumor >10 mm in size. In addition to tumor size, it is important to focus on the intra-tumor components and the layers of the gall bladder to detect the malignant transformation of GBPs. Among the several radiological interventions available, EUS is superior in differentiating the double-layered structure of the GB wall and provides higher resolution for small polypoid lesions. Jae Hee Cho et al. investigated 88 patients who underwent EUS for polyps of <20 mm; 33 of these were neoplastic polyps and they reported that the presence of hypo-echoic foci was a predictor in multivariate analysis [14]. EUS findings that are indicative of malignancy include a broad base, a size of >10 mm, laminar rupture, a tendency to increase in size, and the existence of hypo-echoic foci [11], [14]. Focusing on the adenomatous nature of the polyps is critical to detect malignant transformation; however, as the tumors grow larger, the internal echogenicity becomes parenchymatous, making it difficult to distinguish between adenomas and carcinomas. In our case, EUS of this polyp demonstrated a parenchymal-like internal echogenic pattern. There was no laminar rupture or other findings suggestive of malignancy or invasive cancer; however, the polyps showed an increase in size of 4 mm in 6 years.

Previous studies have attempted to identify the predictable ultrasonographic features of premalignant and malignant polypoidal gallbladder lesions [15], [16], [17]. Considering that the rate of adenocarcinoma in adenoma is as high as 3–8%, making a precise diagnosis of GB adenoma is valuable [18]. Contrast-enhanced ultrasound provides significant findings such as homogenous echogenicity on peak-time enhancement, a continuous gallbladder wall, and an eccentric enhancement pattern, which are indicators of gallbladder adenoma [19]. Sun et al. revealed that GBPLs with adenomas >1.15 cm in size, intralesional blood flow, and absence of coexisting cholecystitis had a relatively high likelihood of malignancy in polypoid lesions [20]. Moreover, despite vigorous efforts to standardize these US features, inter-observer variability remains a major limitation in their use for differential diagnosis. Limitations of US findings for GBPLs suggest that artificial intelligence (AI) may be an effective medical aid [21]. Jeong et al. reported that a deep learning-based decision support system (DL-DSS) can reduce the gap between reviewers with different experience levels. In the subgroup analysis, human reviewers over-diagnosed polyps measuring 10 mm or more as neoplastic polyps, and this led to unnecessary cholecystectomies, which could be reduced by lowering the false-positive rate, by using DL DSS.

Pathologically, the disease is classified into three types: pyloric, intestinal, and biliary, or four types, including the foveolar type. Of these, the pyloric gland type is the most common, accounting for >70% of all gallbladder adenomas. The Armed Forces Institute of Pathology also states that pyloric adenomas have a tubular morphology, with intra-adenomatous carcinoma and invasive carcinoma in 7% of patients [22], [23]. Owing to the presence of structural and cellular atypia caused by inflammation, determining the benign or malignant nature of pyloric adenoma and small GBC with intracholecystic papillary-tubular neoplasm, gastric pyloric, and simple mucinous type from H&E stained specimens alone, is difficult. In this case, p53 was partially positive, which helped in the diagnosis. MUC1 staining tends to be positive in areas of high atypia and has been reported to be positive in areas of low atypia too, and MUC5AC and MUC6 staining have been reported to have inconsistent staining patterns and atypia [23], [24].

## 4 Conclusion

Considering the surgical stress of cholecystectomy and the malignant potential of GBC, preceding surgery would be acceptable. A combined analysis of EUS with other radiological modalities would make the earlier diagnosis of GBPLs possible.

## Consent for publication

Written informed consent was obtained from the patient for the publication of this case report and accompanying image.

## Ethics approval

The ethics committee of our institution approved all the procedures used in this study.

## Funding

There is no funding body in the design of the study, collection, analysis, and interpretation of data and in writing the manuscript.

## Guarantor

Tomoyuki Abe

## Research registration number

None.

## CRediT authorship contribution statement

Nao Kitasaki is the first author, and Tomoyuki Abe is a corresponding author.

Nao Kitasaki, Tomoyuki Abe and Akihiko Oshita contribute to the study concept and design.

Nao Kitasaki, Tomoyuki Abe, Shuji Yonehara, Toshio Noriyuki and Masahiro Nakahara contribute to data collection, data analysis and interpretation.

Nao Kitasaki, Tomoyuki Abe, Tsuyoshi Kobayashi, Keiji Hanada and Hideki Ohdan contribute to the writing of the paper.

## Declaration of competing interest

The authors declare that they have no competing interests.
